# Differences in kidney prognosis between congenital and infantile nephrotic syndrome

**DOI:** 10.1007/s00467-025-06735-z

**Published:** 2025-03-17

**Authors:** Yuta Inoki, Tomoko Horinouchi, Shuhei Aoyama, Yuka Kimura, Yuta Ichikawa, Yu Tanaka, Chika Ueda, Hideaki Kitakado, Atsushi Kondo, Nana Sakakibara, Koichi Kamei, Riku Hamada, Naoya Fujita, Yoshimitsu Gotoh, Yoshitsugu Kaku, Kei Nishiyama, Takayuki Okamoto, Yukiko Toya, Tomohiko Yamamura, Shingo Ishimori, China Nagano, Kandai Nozu

**Affiliations:** 1 Department of Pediatrics, Kobe University Graduate School of Medicine, 7-5-1 Kusunoki-Cho, Chuo, Kobe, Hyogo 650-0017 Japan; 2 Division of Nephrology and Rheumatology, National Center for Child Health and Development, Setagaya-Ku, Tokyo Japan; 3 Department of Nephrology and Rheumatology, Tokyo Metropolitan Children’s Medical Center, Fuchu, Tokyo Japan; 4 Department of Pediatric Nephrology, Aichi Children’s Health and Medical Center, Obu, Aichi Japan; 5 Department of Pediatric Nephrology, Japanese Red Cross Aichi Medical Center Nagoya Daini Hospital, Nagoya, Aichi Japan; 6 Department of Nephrology, Fukuoka Children’s Hospital, Fukuoka, Japan; 7 Department of Pediatrics, Graduate School of Medical Sciences, Kyushu University, Fukuoka, Japan; 8 Department of Pediatrics, Hokkaido University Graduate School of Medicine, Sapporo, Hokkaido Japan; 9 Department of Pediatrics, Iwate Medical University, Morioka, Iwate Japan

**Keywords:** Congenital nephrotic syndrome, Infantile nephrotic syndrome, Pathogenic variants, Kidney prognosis, Genotype–phenotype correlation

## Abstract

**Background:**

More than half of patients with congenital nephrotic syndrome (CNS) or infantile nephrotic syndrome (infantile NS) have a monogenic aetiology. This study aimed to clarify differences in the clinical course, genetic background, and genotype–phenotype correlation between CNS and infantile NS.

**Methods:**

We enrolled patients who were diagnosed with CNS or infantile NS and referred to our hospital for genetic analysis and investigated the clinical characteristics and genetic background of patients with identified causative genes.

**Results:**

Among 74 patients enrolled, disease-causing genetic variants were detected in 50 patients. The median age for developing kidney failure in the genetic CNS (*n* = 33) and genetic infantile NS (*n* = 17) groups with monogenic variants was 13.2 and 19.0 months, respectively (*P* = 0.13). The age at developing kidney failure was significantly earlier in CNS patients with genes other than *NPHS1* than in CNS patients with *NPHS1* variants (1.0 vs. 31.0 months; *P* < 0.001). In patients with pathogenic variants other than *NPHS1*, there was a significant difference in the age at developing kidney failure between CNS and infantile NS patients (1.0 vs. 15.0 months; *P* < 0.001). Of patients with *NPHS1* variants, no infants with NS had any truncating variants or developed kidney failure during follow-up.

**Conclusions:**

The onset of CNS or infantile NS affects the kidney prognosis in patients with genetic nephrotic syndrome. Among patients with pathogenic variants in the same gene, patients with infantile NS may have a milder genotype and better prognosis than those with CNS.

**Graphical abstract:**

**Figure:**
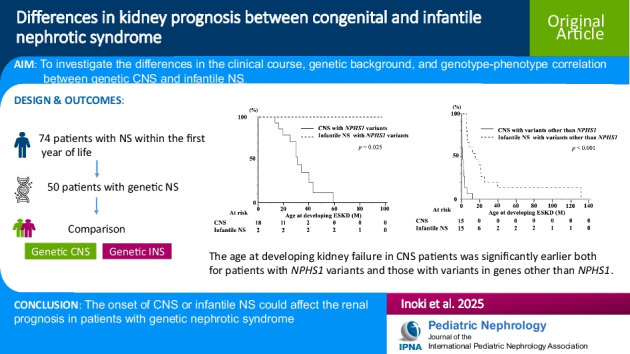
A higher-resolution version of the Graphical abstract is available as Supplementary information

**Supplementary Information:**

The online version contains supplementary material available at 10.1007/s00467-025-06735-z.

## Introduction

Nephrotic syndrome (NS) is the most common glomerular disease in paediatric patients, characterised by severe proteinuria, hypoalbuminaemia, and oedema. Congenital nephrotic syndrome (CNS) is defined as the onset of NS within the first 3 months after birth, while infantile nephrotic syndrome (infantile NS) is defined as the onset of NS between 4 and 12 months after birth [1, 2]. More than half of patients with CNS or infantile NS have a monogenic aetiology that affects the structural and functional integrity of the glomerular filtration barrier [3–6]. Patients with genetic CNS or infantile NS are typically resistant to immunosuppressive therapy and have an unfavourable kidney prognosis [5, 7–9].

The most common disease-causing gene in patients with CNS or infantile NS is *NPHS1*, which is more prevalent in the Finnish population than in any other ethnic group [10, 11]. Other relevant genes include *WT1*, *NPHS2*, *PLCE1*, and *LAMB2* [3–5, 7, 12]. In a European cohort study, 66% of patients with CNS or infantile NS had pathogenic, disease-causing variants in one of the four genes of *NPHS1*, *NPHS2*, *WT1*, and *LAMB2* [3]. Furthermore, genotype–phenotype correlations have been reported for several genes [5, 7, 13–18].

A recent large cohort study in North America showed that patients with CNS had a more severe clinical course, requiring more frequent albumin infusions and earlier nephrectomies, than those with infantile NS [19]. However, few studies have revealed the clinical background, disease-causing genes, and genotype–phenotype correlations in patients with CNS or infantile NS and apparent monogenic variants. We previously reported that the identified rate of genetic variants in Japanese patients with severe proteinuria using next-generation sequencing (NGS) was 85% in patients with CNS and 53% in patients with infantile NS [20].

In this study, we investigated differences in the clinical presentation and kidney prognosis between monogenic CNS and infantile NS. We also assessed the genotype–phenotype correlations in this cohort.

## Methods

### Study design and patient population

This retrospective, observational study included patients diagnosed with NS within the first year of life referred to Kobe University Graduate School of Medicine for genetic analysis between January 2016 and February 2023. Most patients were followed up at other hospitals in Japan. Their management, including the indication for biopsy, the use of drugs, the indication for nephrectomy, and the timing of genetic analysis varied and depended on the treating physician’s judgement (e.g. patients who achieved remission with an immunosuppressive agent did not undergo genetic analysis) according to the clinical guidelines [21]. The final follow-up date was 1 February 2024.

### Definitions

Nephrotic syndrome was defined as nephrotic-range proteinuria (urine protein–creatinine ratio ≥ 2 g/g in the morning urine) and hypoalbuminaemia (serum albumin concentration < 3.0 g/dL) according to recent clinical guidelines [21, 22]. CNS was defined as the onset of NS within 3 months of birth, while infantile NS was defined as the onset between 4 and 12 months of age.

Remission was defined as a urine protein–creatinine ratio < 0.2 g/g in a spot urine test or a dipstick-negative result for proteinuria for ≥ 3 consecutive days. The age at the development of kidney failure was defined as the time of initiating kidney replacement therapy. Regular albumin administration was defined as the required administration of albumin at least once a week for more than 1 month.

### Data collection and analysis

Clinical data were obtained from patient medical records at the time of genetic analysis from all 35 centres. Follow-up data were obtained from the attending physicians at the 25 facilities. Clinical data included sex, initial symptoms, age at onset of NS, presence of oedema, presence of a large placenta, age at discontinuation of regular albumin administration, age at nephrectomy, age at development of kidney failure, and age at the last follow-up. Laboratory data and pathological findings were also extracted from medical records.

All patients were analysed by NGS as detailed below, and the correlation between the type of genetic variants detected and the age of NS onset was investigated. The patients’ characteristics, including clinical symptoms, pathological findings, and genetic variants, were compared between patients with CNS and those with infantile NS. Additionally, the age at which patients developed kidney failure was compared between the two groups using the Kaplan–Meier method.

### Genetic analysis

As we previously reported [20], genomic DNA was isolated from peripheral blood leucocytes of patients and their family members. Targeted sequencing by NGS was performed for genes associated with inherited kidney diseases (Supplementary Table S1). NGS samples were prepared using the Haloplex and Sure Select target enrichment system kit (Agilent Technologies, Santa Clara, CA, USA) according to the manufacturer’s instructions. All indexed DNA samples were amplified by polymerase chain reaction and sequenced using the MiSeq platform (Illumina, San Diego, CA, USA). Sequence data were analysed using SureCall software (version 4.0, Agilent Technologies). SureCall pair analysis was used to determine copy number changes in experimental samples relative to a reference sample without copy number changes. Additional custom array comparative genomic hybridization was performed when the identified exons (more than two exons) in a single patient showed deletions consistent with the patient’s clinical presentation.

When a suspected splice site variant was detected, we also performed a ‘minigene’ splicing assay in vitro or mRNA analysis to assess pathogenicity, as previously reported [23].

### Variant evaluation

The pathogenicity of each variant was classified according to the American College of Medical Genetics (ACMG) consensus guidelines [24]. The following prediction tools were used for analyses in silico: Sorting Intolerant from Tolerant (SIFT; http://sift.bii.a-star.edu.sg), PolyPhen2 (http://genetics.bwh.harvard.edu/pph2/), Mutation Taster (https://www.mutationtaster.org/), and Align GVGD (http://agvgd.iarc.fr).

Patients were diagnosed with monogenic NS when they had one (AD inheritance) or two (AR inheritance) variants of ‘likely pathogenic’ or ‘pathogenic’ according to the ACMG criteria. Variants of ‘uncertain significance’ or ‘likely benign’ were not considered to be disease-causing variants.

### Genotype–phenotype correlation

In patients with pathogenic variants in *NPHS1*, *WT1*, or *LAMB2*, the severity of each variant was defined to assess the genotype–phenotype correlation. *NPHS1* and *LAMB2* truncating (nonsense, frameshift, and large deletion) variants were defined as ‘severe’, while other variants were defined as ‘mild’ [5, 7, 14, 18, 25, 26]. *WT1* exon 8 or 9 missense variants in DNA-binding sites were defined as ‘severe’, while the other variants, including Cys2-His2 Zinc finger structure sites, were defined as ‘mild’ [15, 16, 27].

In patients with pathogenic variants in *NPHS1* or *LAMB2*, the age of onset of kidney failure was compared between those with biallelic, monoallelic, and no severe variants. In patients with pathogenic variants in *WT1*, the age of onset of kidney failure was compared between patients with severe and mild variants.

### Statistical analyses

Data were expressed as the median with interquartile range (IQR) for continuous variables or the percentage for categorical variables. A Mann–Whitney *U* test was used to compare continuous variables, and a Fisher exact test was used to compare categorical variables. The Kaplan–Meier method and the log-rank test were used to compare the occurrence of events (median age of developing kidney failure), which was expressed as the median survival time. *P* < 0.05 was considered significant. The analyses were performed using JMP version 14.0 (SAS Institute Japan, Tokyo, Japan).

## Results

### Baseline characteristics

During the study period, 74 patients who developed NS within the first year of life were identified, 27 of whom were included in previously reported cohorts by our group [16, 17, 20, 28, 29]. Of these 74 patients, disease-causing variants were detected in 50 patients; 33 of 42 (78.6%) patients had CNS, and 17 of 32 (53.1%) patients had infantile NS. The clinical characteristics of the 50 patients from 47 families diagnosed with monogenic NS are shown in Table 1. The median age at diagnosis was 0.1 months (IQR, 0.1–0.6 months) for CNS and 7.0 months (IQR, 4.9–10.6 months) for infantile NS. The incidence of oedema at diagnosis was 80.7% in patients with CNS and 62.5% in patients with infantile NS, with no significant difference between the two groups (*P* = 0.29).

**Table 1:** Characteristics of patients who were identified as having disease-causing genetic variants

	All	Genetic NS	Genetic CNS	Genetic infantile NS
Total number	74	50	33	17
Male sex	38 (51.4)	24 (48.0)	17 (51.5)	7 (41.2)
Age at diagnosis (months)	2.0 [0.1–7.0]	0.6 [0.1–5.2]	0.1 [0.1–0.6]	7.0 [4.9–10.6]
Age at last follow-up (years)		5.5 [2.5–7.7]	3.5 [2.0–6.7]	7.5 [5.6–10.1]
Initial indication				
Blood test or urine test	30 (44.1)	23 (46.0)	15 (45.6)	8 (47.1)
Oedema	32 (47.1)	21 (42.0)	13 (39.4)	8 (47.1)
Poor weight gain	2 (2.9)	2 (4.0)	1 (3.0)	1 (5.9)
Oliguria	4 (5.9)	4 (8.0)	4 (12.1)	0 (0.0)
Clinical findings				
Serum albumin (g/dL)	1.2 [0.9–1.9]	1.2 [0.7–1.7]	1.0 [0.7–1.7]	1.2 [0.9–1.9]
Oedema at diagnosis	47 (77.0)	35 (74.5)	25 (80.7)	10 (62.5)
Large placenta	26 (57.8)	25 (50.0)	24 (72.7)*	1 (5.9)*
Type of gene				
*NPHS1*		20	18 (54.6)	2 (11.8)
*WT1*		13	5 (15.2)	8 (47.1)
*LAMB2*		7	7 (21.2)	0 (0)
*LAMA5*		3	1 (3.0)	2 (11.8)
*ARHGDIA*		2	2 (6.1)	0 (0)
*TTC21B*		1	0 (0.0)	1 (5.9)
*PODXL*		1	0 (0.0)	1 (5.9)
*COQ6*		1	0 (0.0)	1 (5.9)
*TRPC6*		1	0 (0.0)	1 (5.9)
*PLCE1 (NPHS3)*		1	0 (0.0)	1 (5.9)
Pathological findings				
MCN	5 (10.6)	2 (4.0)	1 (5.0)	1 (6.7)
FSGS	15 (31.9)	10 (20.0)	3 (15.0)	7 (46.7)
DMS	18 (38.3)	17 (34.0)	10 (50.0)	7 (46.7)
Others	9 (19.1)	6 (12.0)	6 (30.0)	0 (0)

Data are expressed as the median [interquartile range] or number (%)

*CNS* congenital nephrotic syndrome, *infantile NS* infantile nephrotic syndrome, *MCN* minimal change nephropathy, *FSGS* focal segmental glomerulosclerosis, *DMS* diffuse mesangial sclerosis

^*^P < 0.001

**Fig. 1:**
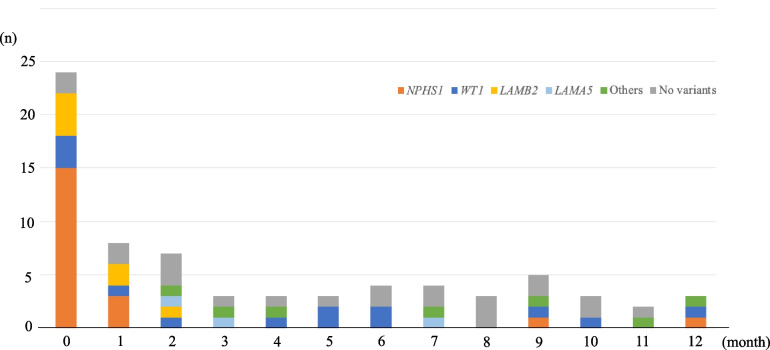
Age at diagnosis of nephrotic syndrome with detected pathogenic variants in disease-causing genes

The types of disease-causing genes are shown in Table 1. *NPHS1*, *WT1*, and *LAMA5* were detected in patients with CNS and infantile NS. In the CNS group, all patients with *NPHS1* variants, 4 of 5 (80%) patients with *WT1* variants, and 6 of 7 (85.7%) patients with *LAMB2* variants were diagnosed within the first month of life (Fig. 1). Most patients with *NPHS1* variants presented with NS within the first 2 months of life, whereas those with *WT1* variants manifested throughout infancy. In addition to major genes, rare gene variants, such as *LAMA5* (*n* = 3), *ARHGDIA* (*n* = 2), *COQ6*, *TRPC6*, *TTC21B,* and *PODXL* (*n* = 1 each), were detected in our cohort.

A kidney biopsy was performed in 20 patients with CNS and in 15 with infantile NS (Table 1). Light microscopy showed that 10 (50.0%) patients with CNS, and 7 (46.7%) patients with infantile NS had diffuse mesangial sclerosis, with no significant difference between the two groups (*P* = 1.0). Focal segmental glomerulosclerosis was present in three (15.0%) patients with CNS and in seven (46.7%) patients with infantile NS. Nonspecific tubulointerstitial changes, including tubular dilatation, were present in four patients with *NPHS1* variants.

### Clinical course

Table 2 shows the kidney and survival prognosis. All patients who underwent nephrectomy received a unilateral nephrectomy rather than a bilateral nephrectomy. The incidence of regular albumin administration and nephrectomy before kidney failure was significantly higher in patients with CNS than in those with infantile NS (*P* = 0.049 and *P* = 0.018, respectively). Most patients with CNS and *NPHS1* variants required regular albumin infusion or nephrectomy to reduce regular albumin infusion, whereas infants with NS caused by *NPHS1* variants did not require these treatments (Table 3).

**Table 2:** The clinical course of patients with CNS and those with infantile NS

	Total	CNS	Infantile NS
Total number	50	33	17
Regular albumin administration	16 (34.0)	14 (45.2)^§^	2 (12.5)^§^
Median age at discontinuation of regular albumin administration (months) [IQR]	13.2 [4.6–16.8]	10.1 [4.6–16.8]	18.9 [16.8–21.0]
Immunosuppressive agent	8 (16.0)	0 (0)^‖^	8 (47.1)^‖^
Prednisolone	3 (6.0)	0 (0)	3 (17.7)
Prednisolone + cyclosporin A	5 (10.0)	0 (0)	5 (29.4)
A unilateral nephrectomy before kidney failure	13 (27.1)	12 (38.7)*	1 (5.9)*
Kidney failure	40 (80.0)	26 (78.8)	(82.4)
Median age at development of kidney failure (months) [95% CI]	15.0 [5.8–25.2]	13.2 [2.0–30.0]	19.0 [6.0–40.0]
Remission	1 (2.1)	0 (0)	1 (5.9)
Death	5 (10.9)	3 (10.0)	2 (12.5)

Data are expressed as the median [IQR], median [95% CI], or number (%)

*IQR* interquartile range, *CI* confidence interval, *CNS* congenital nephrotic syndrome, *Infantile NS* infantile nephrotic syndrome

^*^P = 0.018

^§^*P* = 0.049

^‖^*P* < 0.001

**Table 3:** Comparison of the clinical course between patients with CNS and those with infantile NS according to the type of gene

	*NPHS1*		*WT1*	
	CNS	Infantile NS	CNS	Infantile NS
Number of patients	18	2	5	8
Male sex	11 (61.1)	2 (100)	1 (20.0)	2 (25.0)
Median age at diagnosis (month) [IQR]	0.1 [0.1–0.1]	10.2 [8.5–11.9]	0.2 [0.1–1.2]	6.0 [4.8–9.8]
Oedema at diagnosis	11 (68.8)	2 (100)	5 (100)	4 (57.1)
Pathological findings	11	1	5	8
MCN	1	1	0	0
FSGS	3	0	0	2
DMS	2	0	5	6
Others	5	0	0	0
Regular albumin administration	14 (87.5)	0 (0.0)	0 (0)	1 (12.5)
Unilateral nephrectomy before kidney failure	12 (75.0)	0 (0.0)	0 (0)	0 (0)
Kidney failure	11 (61.1)	0 (0.0)	5 (100)	8 (100)
Median age at development of kidney failure (months) [95% CI]	31.0 [19.5–43.2]	–	0.5 [0.4–2.0]^‖^	9.1 [5.0–21.0]^‖^
Death	1 (6.7)	0 (0.0)	0 (0)	1 (12.5)

Data are expressed as the median [IQR], median [95% CI], or number (%). This table included two patients with a missense variant with ‘uncertain significance’ according to the American College of Medical Genetics criteria

*IQR* interquartile range, *CI* confidence interval, *CNS* congenital nephrotic syndrome, *Infantile NS* infantile nephrotic syndrome, *MCN* minimal change nephropathy, *FSGS* focal segmental glomerulosclerosis, *DMS* diffuse mesangial sclerosis

^‖^*P* < 0.001

### Kidney prognosis

**Fig. 2:**
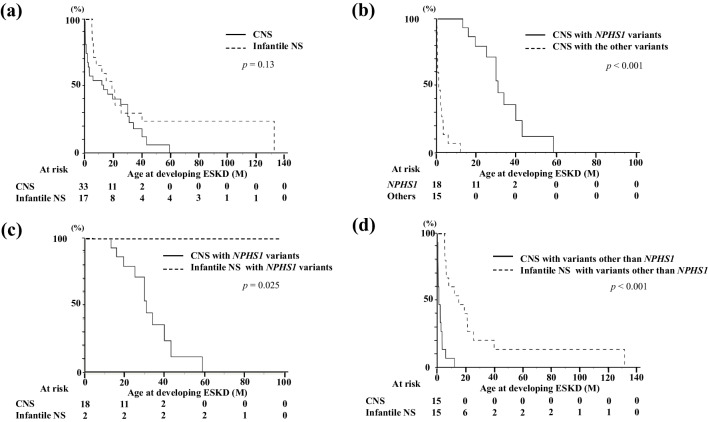
Probability of developing kidney failure in patients with pathogenic variants. **a** Comparison of the age at which kidney failure develops between patients with CNS and those with infantile NS. No significant difference in age was observed between the two groups (13.2 vs. 19.0 months; *P* = 0.13). **b** Comparison of the age at which kidney failure develops between patients with CNS with *NPHS1* variants and patients with CNS with other variants. A significant age difference was observed between the two groups (1.0 vs. 31.0 months; *P* < 0.001). **c** Comparison of the age at which kidney failure develops between patients with CNS with *NPHS1* variants and patients with infantile NS with *NPHS1* variants. A significant age difference was observed between the two groups (31.0 vs. − months; *P* = 0.025). **d** Comparison of the age at developing kidney failure between patients with CNS with pathogenic variants other than *NPHS1* and patients with infantile NS with pathogenic variants other than *NPHS1*. A significant age difference was observed between the two groups (1.0 vs 15.0 months; *P* < 0.001). CNS, congenital nephrotic syndrome; infantile NS, infantile nephrotic syndrome

Compared with the cohort of 50 patients with a definite genetic diagnosis, there was no significant difference in the age of the development of kidney failure between patients with CNS and those with infantile NS (13.2 vs. 19.0 months; *P* = 0.13) (Fig. 2a). Based on previous studies [7, 9], the age of developing kidney failure was compared between CNS patients with *NPHS1* and those with the other gene variants. CNS patients with *NPHS1* variants developed kidney failure significantly later than those with the other gene variants (31.0 vs. 1.0 months; *P* < 0.001) (Fig. 2b). Based on these results, kidney prognosis was compared separately for *NPHS1* and other variants. Patients with CNS progressed to kidney failure earlier than those with infantile NS regarding *NPHS1* variants, as well as when all non-*NPHS1* variants were combined (31.0 vs. − months; *P* = 0.025, and 1.0 vs. 15.0 months; *P* < 0.001, respectively) (Fig. 2c and d). In patients with *WT1* variants (*n* = 13), those with CNS developed kidney failure earlier than those with infantile NS (0.5 vs. 9.1 months of age; *P* < 0.001) (Table 3).

### Genotype–phenotype correlation

The genotype–phenotype correlation is shown in Table 4. Patients with *NPHS1* biallelic severe variants and those with *NPHS1* monoallelic severe variants were not significantly different in age at the development of kidney failure (31.0 vs. 25.2 months; *P* = 0.29). By contrast, patients without severe variants did not develop kidney failure during the follow-up period. In patients with *NPHS1* variants, all but one with CNS had severe variants in at least one allele, whereas those with infantile NS had no severe variants.

**Table 4:** Genotype–phenotype correlations in patients with pathogenic variants in *NPHS1*, *WT1*, or *LAMB2*

	*NPHS1*			*WT1*		*LAMB2*		
	Biallelic truncating variants	Monoallelic truncating variant	No truncating variants	Severe variant*^1^	Mild variant*^1^	Biallelic truncating variants	Monoallelic truncating variant	No truncating variants
Patient number								
CNS	6	11	1	5	0	4	2	1
Infantile NS	0	0	2	3	5	0	0	0
Kidney failure	5 (83.3)	6 (54.5)	0 (0.0)	8 (100)	5 (100)	4 (100)	2(100)	1 (100)
Median age at kidney failure (months) [95% CI]	25.2 [13.2–43.2]^§^	31.0 [19.5–59.0]^§^	–	1.5 [0.4–5.3]^†^	15.0 [5.0–132.0]^†^	0.7 [0.3–5.8]	1.3 [0.1–2.5]	3.3

Data are expressed as the median [95% CI] or number (%)

*CI* confidence interval, *CNS* congenital nephrotic syndrome, *Infantile NS* infantile nephrotic syndrome

^*^^1^A severe variant was defined as a variant in the DNA-binding site

^§^*P* = 0.29

^†^*P* = 0.006

In patients with *WT1* pathogenic variants, 11 had missense variants in exon 8 or 9, 1 had a variant in exon 1, and 1 had a variant in intron 9; 8 of 11 patients with variants in exon 8 or 9 had variants in DNA-binding sites and were classified as having a ‘severe’ genotype. Patients with severe variants developed kidney failure significantly earlier than those without severe variants (aged 1.5 vs. 15.0 months; *P* = 0.006). All patients with CNS and 37.5% (3/8) of those with infantile NS had severe variants. In patients with infantile NS, 3 had exon 8 or 9 variants in Cys2-His2 Zinc finger structure sites, which have been reported to be associated with a relatively severe phenotype [16].

In patients with *LAMB2* variants, all patients with CNS developed kidney failure at a median age of 1.0 month. There was no significant difference in the age of kidney failure between patients with biallelic, monoallelic, or no severe variants.

## Discussion

In the present study, disease-causing genetic variants were detected in 80.1% of patients with CNS and 56.2% with infantile NS. CNS patients with *NPHS1* variants developed kidney failure significantly later than those with the other variants. Patients with variants in *NPHS1* or non-*NPHS1* had milder disease when they manifested as infantile NS, while those with CNS had more severe disease. Furthermore, patients with *NPHS1* or *WT1* variants showed genotype–phenotype correlations, and the infantile NS group had more patients with mild variants.

The incidence of disease-causing genes varies by region and race, while the identification rate may be affected by criteria for genetic testing and methodology (Sanger sequencing or NGS). Previous studies reported that 72–90% of patients with CNS and 36–77% of those with infantile NS had a monogenic aetiology [3–7, 16, 19, 30], which is consistent with our study. The difference in the identification rate in patients with infantile NS between studies may be due to differences in the criteria and timing of genetic testing. In cohort studies conducted in regions where genetic testing can be performed at an early stage, a certain proportion of infantile-onset idiopathic NS cases that eventually achieve remission tend to be included. This characteristic may affect the identification rate of genetic abnormalities. In Japan, a nationwide cross-sectional survey reported that 86.6% of patients with NS onset before 1 year of age had undergone genetic testing [9]. Therefore, our results also suggest a similar rate of genetic testing participation.

In our cohort, 40% of patients in both CNS and infantile NS groups were first diagnosed via blood or urine tests, which were as common as oedema. The findings of an absence of oedema on diagnosis in the face of significant hypoalbuminemia in some CNS and infantile NS patients were consistent with previous reports [31]. In the CNS group, some patients were diagnosed after blood or urine tests because of transient tachypnoea of the newborn, possibly caused by hypoalbuminemia from the foetal period [32]. However, some patients with infantile NS who were diagnosed with blood or urine tests underwent these tests because they experienced infections. These infections may have been related to the susceptibility of these patients to infection or because they were more likely to be severely ill owing to the nephrotic state.

In the present study, the kidney prognosis of patients with *NPHS1* variants was better than those with other genetic variants. Consequently, there was no significant difference in the kidney prognosis when we compared the entire cohort of genetic CNS and infantile NS because of the predominance of *NPHS1* variants in patients with CNS. Another study showed that the development of kidney failure was more common in CNS (80%) than in infantile NS (60%), and our results are consistent with these findings [19]. However, the previous study did not differentiate between genetic and non-genetic NS, and some patients did not undergo genetic analysis, possibly skewing the results. Additionally, more than half of the patients with CNS underwent bilateral nephrectomy, which led to earlier onset of kidney failure. Here, no patient had bilateral nephrectomy before kidney replacement therapy, in accordance with the European Reference Network for Kidney Diseases (ERKNet) and the European Society for Paediatric Nephrology (ESPN) guidelines [33]. To our knowledge, this is the first study to compare the kidney prognosis between monogenic CNS and infantile NS under the latest treatment guidelines, focusing on specific causative genes *(NPHS1*, *WT1*, and *LAMB2*).

Previous reports of CNS cases with *NPHS1* variants defined the severe genotype as having biallelic truncating variants and showed no correlation with the kidney prognosis [5, 7]. Our present study also showed no significant difference between patients with or without biallelic truncating variants. However, we found that patients with biallelic or monoallelic truncating (severe) variants in *NPHS1* had a better kidney prognosis than those without truncating variants. Although all but one patient with CNS in this study had at least one truncating variant, a previous report showed that some patients without truncating variants in *NPHS1* developed CNS, which may be because some missense variants have a strong pathogenicity that is equal to truncating variants [25]. This difference between studies suggests that there may be a range in the severity of non-truncating variants, and the variants detected in this study resulted in a milder phenotype with infantile NS onset.

The present study showed that all CNS patients with *WT1* variants had missense variants in the DNA-binding site and developed kidney failure within the first 3 months of life. However, some infantile NS patients with *WT1* variants had missense variants in other sites and showed a milder clinical course. Previous studies showed that patients with *WT1* missense variants in exon 8 or 9 developed kidney failure earlier than those with truncating or splice site variants, and the median age at kidney failure onset ranged from 0.22 to 2.5 years [15, 26, 27, 34]. Moreover, several studies have found that missense variants in the DNA-binding site caused a more severe kidney prognosis than other missense variants, and our findings are consistent with these findings [15, 16]. Our group previously conducted a systematic review of 174 patients with variants in exon 8 or 9 and showed that variants not only in the DNA-binding site but also in Cys2-His2 Zinc finger structure sites resulted in a more severe kidney prognosis than variants in other sites [16]. In our study, patients with variants in Cys2-His2 Zinc finger structure sites showed a severe phenotype and early onset within 1 year, but it was not as severe as that in those with DNA-binding site variants.

The present study has several limitations. First, some data were missing because this was a retrospective study, and there were gaps in the data. Second, the cohort studied was small, which may have resulted in a founder effect and may limit the external validity of our findings. Third, the number of kidney biopsies performed was limited, and no distinctive findings were observed in this study. Finally, the timing of gene analysis depended on the attending physician, which may have affected the incidence of disease-causing genes.

In conclusion, the present study suggests that patients with CNS have a worse kidney outcome than those with infantile NS. This finding is present in patients with *NPHS1* and other gene variants. Additionally, patients with infantile NS may be more likely to have a mild genotype and good kidney prognosis. Further large-scale studies are required to confirm our findings.

## Supplementary Information

Below is the link to the electronic supplementary material.

Graphical abstract (PPTX 251 KB)

Supplementary file2 (DOCX 3210 KB)

## Data Availability

The data underlying this article will be shared upon reasonable request by the corresponding author.
