# Supplementary material for: Differences in kidney prognosis between congenital and infantile nephrotic syndrome
Source: Pediatr Nephrol. 2025 Mar 17;40(8):2539–49. doi: 10.1007/s00467-025-06735-z (PMC12187875; doi:10.1007/s00467-025-06735-z)
Supplement: Supplementary file 1 — Graphical abstract (PPTX 251 KB) [file 467_2025_6735_MOESM1_ESM.pptx]

## Slide 1
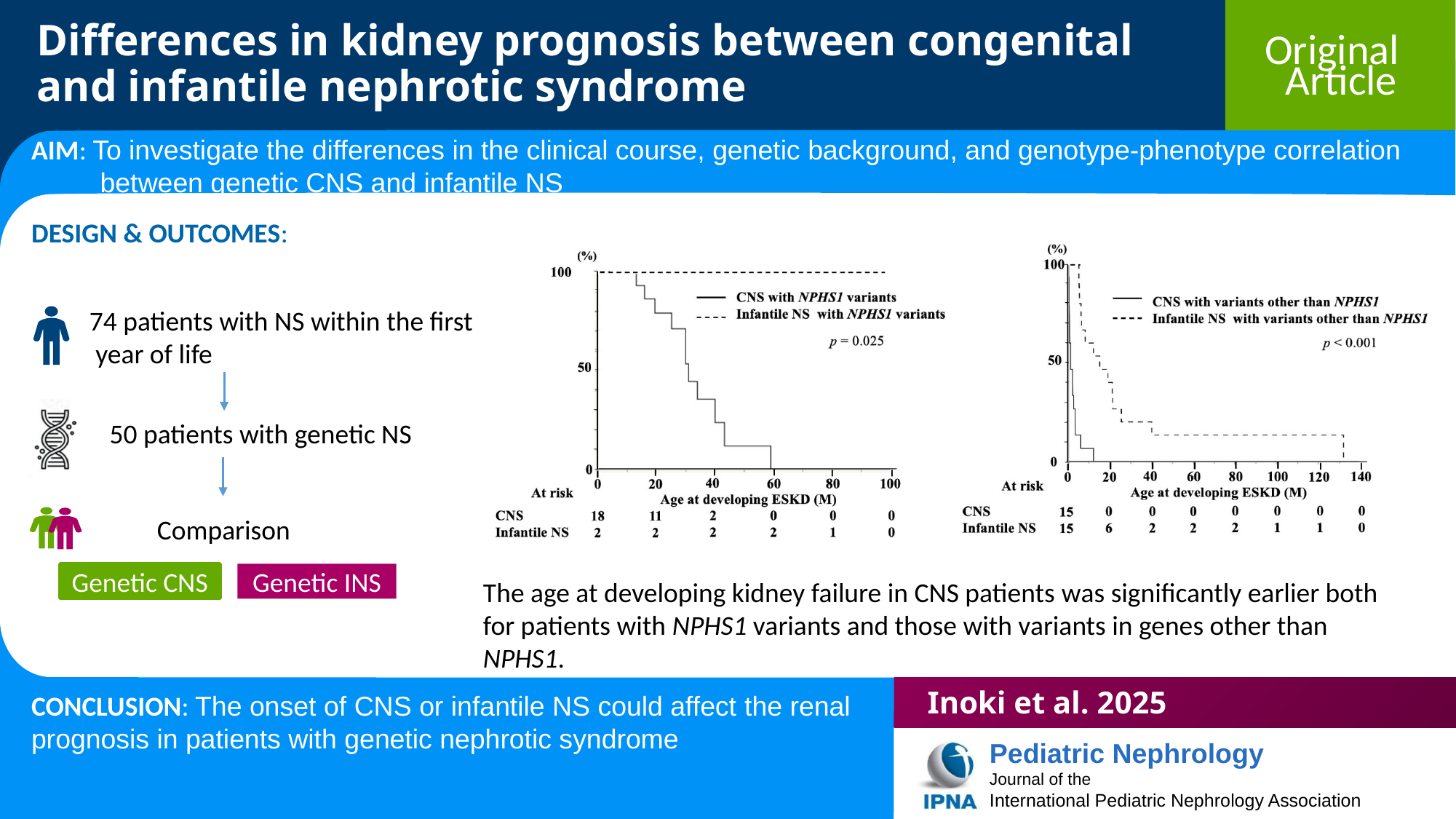

Differences in kidney prognosis between congenital and infantile nephrotic syndrome
AIM: To investigate the differences in the clinical course, genetic background, and genotype-phenotype correlation
 between genetic CNS and infantile NS
DESIGN & OUTCOMES:
74 patients with NS within the first
 year of life
50 patients with genetic NS
Comparison
Genetic INS
Genetic CNS
The age at developing kidney failure in CNS patients was significantly earlier both for patients with NPHS1 variants and those with variants in genes other than NPHS1.
Inoki et al. 2025
CONCLUSION: The onset of CNS or infantile NS could affect the renal prognosis in patients with genetic nephrotic syndrome
