# Supplementary material for: Differences in kidney prognosis between congenital and infantile nephrotic syndrome
Source: Pediatr Nephrol. 2025 Mar 17;40(8):2539–49. doi: 10.1007/s00467-025-06735-z (PMC12187875; doi:10.1007/s00467-025-06735-z)
Supplement: Supplementary file 2 — Supplementary file2 (DOCX 3210 KB) [file 467_2025_6735_MOESM2_ESM.docx]

Supplementary data

**Differences in kidney prognosis between congenital and infantile nephrotic syndrome**

Yuta Inoki ^1^, Tomoko Horinouchi ^1^*, Shuhei Aoyama ^1^, Yuka Kimura ^1^, Yuta Ichikawa ^1^, Yu Tanaka ^1^, Chika Ueda ^1^, Hideaki Kitakado ^1^, Atsushi Kondo ^1^, Nana Sakakibara ^1^, Koichi Kamei ^2^, Riku Hamada ^3^, Naoya Fujita ^4^, Yoshimitsu Gotoh ^5^, Yoshitsugu Kaku ^6^, Kei Nishiyama ^7^, Takayuki Okamoto ^8^, Yukiko Toya ^9^, Tomohiko Yamamura ^1^, Shingo Ishimori ^1^, China Nagano ^1^, Kandai Nozu ^1^

^1^ Department of Pediatrics, Kobe University Graduate School of Medicine, Kobe, Hyogo, Japan

^2^ Division of Nephrology and Rheumatology, National Center for Child Health and Development, Setagaya-ku, Tokyo, Japan

^3^ Department of Nephrology and Rheumatology, Tokyo Metropolitan Children’s Medical Center, Fuchu, Tokyo, Japan

^4^ Department of Pediatric Nephrology, Aichi Children’s Health and Medical Center, Obu, Aichi, Japan

^5^ Department of Pediatric Nephrology, Japanese Red Cross Aichi Medical Center Nagoya Daini Hospital, Nagoya, Aichi, Japan

^6^ Department of Nephrology, Fukuoka Children’s Hospital, Fukuoka, Japan

^7^ Department of Pediatrics, Graduate School of Medical Sciences, Kyushu University, Fukuoka, Japan

^8^ Department of Pediatrics, Hokkaido University Graduate School of Medicine, Sapporo, Hokkaido, Japan

^9^ Department of Pediatrics, Iwate Medical University, Morioka, Iwate, Japan

***Corresponding author**

Tomoko Horinouchi, MD, PhD

Department of Pediatrics, Kobe University Graduate School of Medicine,

7-5-1 Kusunoki-cho, Chuo, Kobe, Hyogo 650-0017, Japan

E-mail: tohori@med.kobe-u.ac.jp

Tel: +81-78-382-5111

Fax: +81-78-382-5050

ORCID: <https://orcid.org/0000-0003-1655-6030>

**Supplemental Table S1**. List of 68 podocyte-related genes included in targeted sequencing analysis within a clinically approved gene panel test developed in our laboratory.

| *ACTN4* | *COQ2* | *ITSN1* | *MAGI2* | *PAX2* | *TPRKB* |
| --- | --- | --- | --- | --- | --- |
| *ADCK4* | *COQ6* | *ITSN2* | *MYH9* | *PDSS2* | *TRIM8* |
| *ANKFY1* | *CRB2* | *KANK1* | *MYO1E* | *PLCE1* | *TRPC6* |
| *ANLN* | *CUBN* | *KANK2* | *NPHS1* | *PODXL* | *TTC21B* |
| *ARHGAP24* | *DLC1* | *KANK4* | *NPHS2* | *PRDM15* | *WDR4* |
| *ARHGDIA* | *EMP2* | *KIRREL1* | *NUP85* | *PTPRO* | *WDR73* |
| *AVIL* | *FAT1* | *LAGE3* | *NUP93* | *SCARB2* | *WT1* |
| *CD2AP* | *GAPVD1* | *LAMA5* | *NUP107* | *SGPL1* | *XPO5* |
| *CKD20* | *GON7* | *LAMB2* | *NUP133* | *SMARCAL1* |  |
| *COL4A3* | *INF2* | *LMNA* | *NUP160* | *TNS2* |  |
| *COL4A4* | *ITGA3* | *LMX1B* | *NUP205* | *TBC1D8B* |  |
| *COL4A5* | *ITGB4* | *MAFB* | *OSGEP* | *TP53RK* |  |

**Supplemental Table S2**. Patient phenotype and genotype

| Patient ID | Sex | Gene | MOI | Age at diagnosis  (months) | Age at ESKD  (months) | Exon | Genome | Amino acids | Type of variant | ACMG criteria | Genotype | Previous publication |
| --- | --- | --- | --- | --- | --- | --- | --- | --- | --- | --- | --- | --- |
| ***NPHS1* (CNS)** |  | NM_004646.4 |  |  |  |  |  |  |  |  |  |  |
| Neph27 | M | *NPHS1* | AR | 0.1 | 40.0 | 19 | c.2515delC | p.(Gln839Argfs*8) | Frameshift | Pathogenic (PVS1, PM2, PP5) | severe | [20] |
|  |  |  |  |  |  | 9 | c.1102C>T | p.(Pro368Ser) | Missense | Likely pathogenic (PM1, PM2, PM3, PP5) | mild |  |
| Neph104 | F | *NPHS1* | AR | 0.1 | 16.0 | 19 | c.2515delC (homo) | p.(Gln839Argfs*8) | Frameshift | Pathogenic (PVS1, PM2, PP5) | severe | [20] |
| Neph104-1 | M | *NPHS1* | AR | 0.1 | 34.0 | 19 | c.2515delC (homo) | p.(Gln839Argfs*8) | Frameshift | Pathogenic (PVS1, PM2, PP5) | severe |  |
| Neph106 | F | *NPHS1* | AR | 0.1 | 59.0 | 19 | c.2515delC | p.(Gln839Argfs*8) | Frameshift | Pathogenic (PVS1, PM2, PP5) | severe | [20] |
|  |  |  |  |  |  | 9 | c.1135C>T | p.(Arg379Trp) | Missense | Likely pathogenic (PM1, PM2, PP3, PP5) | mild |  |
| Neph251 | M | *NPHS1* | AR | 0.1 | 43.2 | 7 | c.772G>T (homo) | p.(Gly258*) | Nonsense | Pathogenic (PVS1, PM2, PM3) | severe | Naïve |
| Neph378 | F | *NPHS1* | AR | 0.1 | 30.0 | 22 | c.2927+14_3110-682del | – |  | Likely pathogenic (PVS1, PM2) | severe | Naïve |
|  |  |  |  |  |  | 11 | c.1379G>A | p.(Arg460Gln) | Missense | Likely pathogenic (PM1, PM2, PM3, PP5, BP4) | mild |  |
| Neph387 | F | *NPHS1* | AR | 0.1 | 30.1 | 19 | c.2515delC | p.(Gln839Argfs*8) | Frameshift | Pathogenic (PVS1, PM2, PP5) | severe | Naïve |
|  |  |  |  |  |  | 11 | c.1379G>A | p.(Arg460Gln) | Missense | Likely pathogenic (PM1, PM2, PM3, PP5, BP4) | mild |  |
| Neph431 | M | *NPHS1* | AR | 0.1 | N/A | 11 | c.1316-29_1440+801del | – |  | Likely pathogenic (PVS1, PM2) | severe | Naïve |
|  |  |  |  |  |  | 16 | c.2177G>A | p.(Gly726Asp) | Missense | Likely pathogenic (PM1, PM2, PM3, PP3) | mild |  |
| Neph460 | M | *NPHS1* | AR | 0.1 | 13.2 | 16 | c.2156_c.2163delTGCACTGC (homo) | p.(Leu719Profs*4) | Frameshift | Pathogenic (PVS1, PM2, PM3, PP5) | severe | Naïve |
| Neph517 | M | *NPHS1* | AR | 0.1 | 19.5 | 7 | c.772G>T | p.(Gly258*) | Nonsense | Pathogenic (PVS1, PM2, PM3) | severe | Naïve |
|  |  |  |  |  |  | 9 | c.1135C>T | p.(Arg379Trp) | Missense | Likely pathogenic (PM1, PM2, PP3, PP5) | mild |  |
| Neph519 | F | *NPHS1* | AR | 0.1 | 25.2 | 19 | c.2515delC | p.(Gln839Argfs*8) | Frameshift | Pathogenic (PVS1, PM2, PP5) | severe | Naïve |
|  |  |  |  |  |  | 11 | c.1316-29_1440+801del | – |  | Likely pathogenic (PVS1, PM2) | severe |  |
| Neph534 | M | *NPHS1* | AR | 0.1 | – | 27 | c.3478C>T | p.(Arg1160*) | Nonsense | Pathogenic (PVS1, PM2, PM3, PP5) | severe | Naïve |
|  |  |  |  |  |  | 9 | c.1048T>C | p.(Ser350Pro) | Missense | Likely pathogenic (PM1, PM2, PM3, PP5, BP4) | mild |  |
| Neph647 | F | *NPHS1* | AR | 0.1 | – | 11 | c.2156_c.2163delTGCACTGC | p.(Leu719Profs*4) | Frameshift | Pathogenic (PVS1, PM2, PM3, PP5) | severe | Naïve |
|  |  |  |  |  |  | 11 | c.1379G>A | p.(Arg460Gln) | Missense | Likely pathogenic (PM1, PM2, PM3, PP5, BP4) | mild |  |
| Neph676 | M | *NPHS1* | AR | 0.1 | – | 8 | c.869dupG | p.(Thr291Hisfs*51) | Frameshift | Pathogenic (PVS1, PM2, PM3) | severe | Naïve |
|  |  |  |  |  |  | 11 | c.1379G>A | p.(Arg460Gln) | Missense | Likely pathogenic (PM1, PM2, PM3, PP5, BP4) | mild |  |
| Neph604 | M | *NPHS1* | AR | 0.2 | N/A | IVS6 | c.712+4A>G | (Exon 6 skipping)  306 base spared | Splice-site | Likely pathogenic (PS3, PM2, PM3) | mild | Naïve |
|  |  |  |  |  |  | 11 | c.1379G>A | p.(Arg460Gln) | Missense | Likely pathogenic (PM1, PM2, PM3, PP5, BP4) | Mild |  |
| Neph69 | M | *NPHS1* | AR | 0.8 | 31.0 | 8 | c.869dupG | p.(Thr291Hisfs*51) | Frameshift | Pathogenic (PVS1, PM2, PM3) | severe | [20] |
|  |  |  |  |  |  | 11 | c.1379G>A | p.(Arg460Gln) | Missense | Likely pathogenic (PM1, PM2, PM3, PP5, BP4) | mild |  |
| Neph600 | F | *NPHS1* | AR | 0.8 | – | 19 | c.2515delC | p.(Gln839Argfs*8) | Frameshift | Pathogenic (PVS1, PM2, PP5) | severe | Naïve |
|  |  |  |  |  |  | 27 | c.3478C>T | p.(Arg1160*) | Nonsense | Pathogenic (PVS1, PM2, PM3, PP5) | severe |  |
| Neph424 | M | *NPHS1* | AR | 0.9 | – | 19 | c.2515delC | p.(Gln839Argfs*8) | Frameshift | Pathogenic (PVS1, PM2, PP5) | severe | Naïve |
|  |  |  |  |  |  | 9 | c.1135C>T | p.(Arg379Trp) | Missense | Likely pathogenic (PM1, PM2, PP3, PP5) | mild |  |
| ***NPHS1* (infantile NS)** |  |  |  |  |  |  |  |  |  |  |  |  |
| Neph192 | M | *NPHS1* | AR | 8.5 | – | 9 | c.1102C>T | p.(Pro368Ser) | Missense | Likely pathogenic (PM1, PM2, PM3, PP3, PP5) | mild | [20] |
|  |  |  |  |  |  | 18 | c.2464G>A | p.(Val822Met) | Missense | Likely pathogenic (PM1, PM2, PM3, PP3) | mild |  |
| Neph345 | M | *NPHS1* | AR | 11.9 | – | 16 | c.2207T>C | p.(Val736Ala) | Missense | Likely pathogenic (PM1, PM2, PM3, PP3, PP5) | mild | Naïve |
|  |  |  |  |  |  | IVS23 | c.3166+5G>A | – | Splice-site | Likely pathogenic (PS3, PM2, PM3) | mild |  |
| ***WT1***  **(CNS)** |  | NM_024426.6 |  |  |  |  |  |  |  |  |  |  |
| Neph92 | F | *WT1* | AD | 0.1 | 0.5 | 8 | c.1315C>T | p.(Arg439Cys) | Missense | Likely pathogenic (PM1, PM2, PP3, PP5) | Severe | [20] |
| Neph372 | F | *WT1* | AD | 0.1 | 3 | 9 | c.1400G>A | p.(Arg467Gln) | Missense | Likely pathogenic (PS2, PM2, PP3, PP5) | Severe | Naïve |
| Neph426 | F | *WT1* | AD | 0.2 | 0.4 | 9 | c.1399C>T | p.(Arg467Trp) | Missense | Likely pathogenic (PS2, PM2, PP3, PP5) | Severe | Naïve |
| Neph332 | M | *WT1* | AD | 0.4 | 0.5 | 9 | c.1400G>A | p.(Arg467Gln) | Missense | Likely pathogenic (PS2, PM2, PP3, PP5) | Severe | Naïve |
| Neph107 | F | *WT1* | AD | 1.9 | 2 | 8 | c.1316G>A | p.(Arg439His) | Missense | Pathogenic (PS2, PM1, PM2, PP3, PP5) | Severe | [20] |
| ***WT1* (infantile NS)** |  |  |  |  |  |  |  |  |  |  |  |  |
| Neph7 | F | *WT1* | AD | 3.9 | 5.3 | 9 | c.1399C>T | p.(Arg467Trp) | Missense | Likely pathogenic (PS2, PM2, PP3, PP5) | Severe | [16] |
| Neph243 | F | *WT1* | AD | 4.8 | 5.2 | 9 | c.1399C>T | p.(Arg467Trp) | Missense | Likely pathogenic (PS2, PM2, PP3, PP5) | mild | [16] |
| Neph208 | F | *WT1* | AD | 4.9 | 5 | 8 | c.1349A>G | p.(His450Arg) | Missense | Pathogenic (PS2, PM1, PM2, PP3, PP5) | mild | [16] |
| Neph132 | F | *WT1* | AD | 5.9 | 6.2 | 8 | c.1316G>A | p.(Arg439His) | Missense | Pathogenic (PS2, PM1, PM2, PP3, PP5) | Severe | [20] |
| Neph185 | M | *WT1* | AD | 6 | 12 | IVS9 | c.1447+4C>T | – | Splice-site | Likely pathogenic (PS3, PM2, PP3) | mild | [20] |
| Neph411 | M | *WT1* | AD | 9 | 15 | 1 | c.500T>A | p.(Val167Asp) | Missense | Likely pathogenic (PM2, PM6, PP3, PP5) | mild | [28] |
| Neph421 | F | *WT1* | AD | 10 | 132 | 9 | c.1382G>T | p.(Cys461Phe) | Missense | Pathogenic (PS2, PM2, PM5, PP3, PP5) | mild | Naïve |
| Neph294 | F | *WT1* | AD | 11.9 | 21 | 8 | c.1349A>C | p.(His450Pro) | Missense | Likely pathogenic (PS2, PM2, PP3, PP5) | mild | [16] |
| ***LAMB2*** |  | NM_002292.4 |  |  |  |  |  |  |  |  |  |  |
| Neph23 | F | *LAMB2* | AR | 0.1 | 3.3 | 5 | c.482T>C (homo) | p.(Leu161Pro) | Missense | Likely pathogenic (PM1, PM2, PM6, PP3, PP4, PP5) | mild | [20] |
| Neph58 | M | *LAMB2* | AR | 0.1 | 0.3 | 13 | c.1648C>T | p.(Arg550*) | Nonsense | Pathogenic (PVS1, PM2, PM3, PP4, PP5) | severe | [20] |
|  |  |  |  |  |  | 27 | c.4519C>T | p.(Gln1507*) | Nonsense | Pathogenic (PVS1, PM2, PM3, PP2, PP4, PP5) | severe |  |
| Neph267 | F | *LAMB2* | AR | 0.1 | 0.1 | 29 | c.4904-4905del | p.(Thr1635Argfs*23) | Frameshift | Pathogenic (PVS1, PM2, PM6, PP5) | severe | Naïve |
|  |  |  |  |  |  | 28 | c.4778C>T | p.(Ala1593Val) | Missense | Likely pathogenic (PM2, PM3, PP3, PP4) | mild |  |
| Neph520 | M | *LAMB2* | AR | 0.1 | 1 | 20 | c.2744delA | p.(Asp915Alafs*236) | Frameshift | Pathogenic (PVS1, PM2, PM3) | severe | Naïve |
|  |  |  |  |  |  | 30 | c.5073_5076dupCCAG | p.(Gly1693Profs*8) | Frameshift | Pathogenic (PVS1, PM2, PM3, PP1, PP5) | severe |  |
| Neph524 | F | *LAMB2* | AR | 0.1 | 0.3 | 27 | c.4573C>T (homo) | p.(Gln1525*) | Nonsense | Pathogenic (PVS1, PM2, PM3, PP1, PP4, PP5) | severe | Naïve |
| Neph133 | F | *LAMB2* | AR | 0.2 | 2.5 | 29 | c.4907_4908del | p.(Glu1636ALafs*22) | Frameshift | Pathogenic (PVS1, PM2, PP5) | severe | [20] |
|  |  |  |  |  |  | 28 | c.4616G>A | p.(Alg1539Gln) | Missense | Likely pathogenic (PM1, PM3, PP4, PP5, BP4) | mild |  |
| Neph87 | M | *LAMB2* | AR | 2 | 5.8 | IVS2 | c.250-14_250-3del | – |  | Likely pathogenic (PM2, PM3, PM6) |  | [20] |
|  |  |  |  |  |  | 29 | c.4904_4905del | p.(Thr1635Argfs*23) | Frameshift | Pathogenic (PVS1, PM2, PM6, PP5) | severe |  |
| ***LAMA5*** |  | NM_005560.6 |  |  |  |  |  |  |  |  |  |  |
| Neph236 | M | *LAMA5* | AR | 2.9 | 12 | 68 | c.9232C>T | p.(Arg3078*) | Nonsense | Likely pathogenic (PVS1, PM2) |  | [20] |
|  |  |  |  |  |  | IVS9 | c.1282+1G>A | – | Splice-site | Likely pathogenic (PVS1, PM2) |  |  |
| Neph236-1 | F | *LAMA5* | AR | 4.1 | 40 | 68 | c.9232C>T | p.(Arg3078*) | Nonsense | Likely pathogenic (PVS1, PM2) |  | [20] |
|  |  |  |  |  |  | IVS9 | c.1282+1G>A | – | Splice-site | Likely pathogenic (PVS1, PM2) |  |  |
| Neph 341 | F | *LAMA5* | AR | 7 | 7.2 | 60 | c.8158C>T | p.(Arg2720*) | Nonsense | Pathogenic (PVS1, PM2, PP5) |  | [17] |
|  |  |  |  |  |  | IVS9 | c.1282+1G>A | – | Splice-site | Likely pathogenic (PVS1, PM2) |  |  |
| ***ARHGDIA*** |  | NM_001185077.2 |  |  |  |  |  |  |  |  |  |  |
| Neph577 | M | *ARHGDIA* | AR | 1.7 | 1.9 | 2 | c.153C>G (homo) | p.(Tyr51*) | Nonsense | Likely pathogenic (PVS1, PM2) |  | Naïve |
| Neph375 | M | *ARHGDIA* | AR | 2.9 | 3.2 | 6 | c.553-555delGAC (homo) | p.(Asp185del) | Non-frameshift | Likely pathogenic (PS3, PM2, PP5) |  | Naïve |
| **Others** |  |  |  |  |  |  |  |  |  |  |  |  |
| Neph 38 | F | *PLCE1* (NM_016341.4) | AR | 12 | 25.4 | 3 | c.1477C>T (homo) | p.(Arg493*) | Nonsense | Pathogenic (PVS1, PM2, PP5) |  | Naïve |
| Neph 97 | M | *COQ6* (NM_182476.3*)* | AR | 8.6 | – | 7 | c.782C>T | p.(Pro261Leu) | Missense | Likely pathogenic (PM1, PM2, PM3, PP5) |  | [20] |
|  |  |  |  |  |  |  | heterozygous deletion | – | ­– | – |  |  |
| Neph 291 | M | *TRPC6* (NM_004621.6) | AD | 11.1 | 19 | 2 | c.523C>T | p.(Arg175Trp) | Missense | Likely pathogenic (PM1, PM2, PP3, PP5) |  | [27] |
| Neph326 | M | *TTC21B* (NM_024753.5) | AR | 4 | 21.2 | 4 | c.379G>A | p.(Ala127Thr) | Missense | Likely pathogenic (PM1, PM2, PM3, PP3) |  | [28] |
|  |  |  |  |  |  | 23 | c.2992_2994del | p.(Arg998del) | Non-frameshift | Likely pathogenic (PM2, PM3, PP3, PP4) |  |  |
| Neph 547 | F | *PODXL* (NM_005397.3) | AR | 7 | 8.1 | 3 | c.927G>A | p.(Trp309*) | Nonsense | Pathogenic (PVS1, PM2) |  | Naïve |
|  |  |  |  |  |  | IVS4 | c.1005+6T>G | (Exon 4 skipping)  78 base spared | Splice-site | Likely pathogenic (PS3, PM2, PM3) |  |  |

MOI, mode of inheritance; ESKD, end-stage kidney disease; CNS, congenital nephrotic syndrome; infantile NS, infantile nephrotic syndrome

**Figure Captions**

**Figure. S1** Transcript analysis of three intron variants detected in patients with suspected genetic nephrotic syndrome

1. In Vitro Splicing Assay (minigene assay)
2. Creating DNA fragment by PCR

We amplified genomic DNA extracted from the peripheral leukocytes of patients and controls to construct hybrid minigenes. Primers specific to the target variants in *NPHS1* were designed to complement the ends of the linearized vector, using the In-Fusion primer design tool (HD Cloning Kit; Takara, Shiga, Japan). This approach allowed for the insertion of PCR products into the vector's multiple cloning site, located within an intron between exons A and B. Specifically, we cloned introns 19–21 of *NPHS1* from the Neph345 sample and introns 11–14 of *NPHS1* from the Neph604 sample.

1. Inserting the DNA fragment into vector

We utilized the H492 vector, previously developed in our laboratory, to generate hybrid minigene constructs. This vector is based on the pcDNA3 mammalian expression vector (Invitrogen, Carlsbad, OR) and was designed to mimic in vivo splicing.

1. Transfecting vector (minigene) into human cells and culturing cell to express the RNA of minigene

We performed an In-Fusion cloning reaction, followed by the transfection of the constructs into HEK293T and HeLa cells using Lipofectamine 2000 (Thermo Fisher Scientific). Twenty-four hours post-transfection, total RNA was extracted from the cells using the RNeasy Plus Mini Kit (Qiagen, Hilden, Germany).

1. mRNA analysis

One microgram of total RNA was reverse-transcribed using the RNA to cDNA EcoDry Premix (Double Primed; Takara). PCR was then performed using a forward primer complementary to a segment upstream of exon A (YH307: 5′-ATTACTCGCTCAGAAGCTGTGTTGC-3′) and a reverse primer complementary to a segment downstream of exon B (Y308: 5′-CTGCCAGTTGCTAAGTGAGAGACTT-3′), designed to amplify transcripts exclusively from each minigene. The PCR products were analyzed by electrophoresis on a 1.5% agarose gel and subsequently subjected to Sanger sequencing.

1. c**. 3166+5**G>A variant in *NPHS1* (Neph345) was revealed aberrant splicing in the form of exon 23 skipping by a “minigene” in vitro splicing assay.
2. c.712+4A>G variant in *NPHS1* (Neph604) was revealed aberrant splicing in the form of exon 6 skipping by a “minigene” in vitro splicing assay.
3. c.1005+6T>G variant in *PODXL* (Neph547) was revealed aberrant splicing in the form of exon 4 skipping by mRNA analysis.

Control samples yielded larger bands, corresponding

Pt, patient; WT, wild-type.
